# Pediatric studies and labeling additions required by the U.S. FDA for novel drugs approved from 2011 to 2023: A retrospective cohort study

**DOI:** 10.1371/journal.pmed.1004651

**Published:** 2025-12-17

**Authors:** Rylee McGonigle, Huseyin Naci, Maximilian Siebert, Michelle Ouvina, Alba Gutiérrez-Sacristán, Anita K. Wagner, Florence T. Bourgeois

**Affiliations:** 1 Harvard-MIT Center for Regulatory Science, Harvard Medical School, Boston, Massachusetts, United States of America; 2 Pediatric Therapeutics and Regulatory Science Initiative, Computational Health Informatics Program, Boston Children’s Hospital, Boston, Massachusetts, United States of America; 3 Department of Health Policy, London School of Economics and Political Science, London, United Kingdom; 4 Department of Biomedical Informatics, Harvard Medical School, Boston, Massachusetts, United States of America; 5 Department of Population Medicine, Harvard Medical School and Harvard Pilgrim Health Care Institute, Boston, Massachusetts, United States of America; 6 Department of Pediatrics, Harvard Medical School, Boston, Massachusetts, United States of America; University of Liverpool, UNITED KINGDOM OF GREAT BRITAIN AND NORTHERN IRELAND

## Abstract

**Background:**

The U.S. Food and Drug Administration (FDA) has the authority to require that sponsors conduct pediatric studies for certain new drugs under the Pediatric Research Equity Act (PREA). Here, we evaluate the characteristics and completion of these studies and assess the addition of pediatric-specific evidence generated from these studies into drug labeling.

**Methods and findings:**

We performed a retrospective cohort study of all novel drugs approved by the FDA from 2011 to 2023 with at least one pediatric study requirement issued under PREA. Study status and outcomes were followed through 31 December 2024. We assessed completion of pediatric studies; addition of pediatric prescribing information to drug labels; and deviations from FDA-projected timelines. Of 552 novel drugs approved by the FDA between 2011 and 2023, 179 (32.4%) were subject to pediatric study requirements under PREA. Thirteen were later discontinued, resulting in a final cohort of 166 drugs and 338 pediatric study requirements. About half (51.8%) of the studies assessed efficacy. Among 222 studies with due dates by 31 December 2024, only 24.3% were completed by the original deadline. Over half (56.8%) received extensions of original timelines, by an average of 2.9 years (SD 2.0). At 10 years after drug approval, while 92.0% of studies were expected to have been completed, 59.5% had been completed. Of the 117 drugs with studies due by 31 December 2024, 54.7% (*n* = 64) had pediatric labeling updated with results from required studies. The mean time to addition of pediatric approval was 5.7 years (SD 2.6), whereas labeling additions reflecting lack of pediatric safety or benefit took an average of 8.3 years (SD 3.3) (*p* < 0.001). While 90.4% of drugs were expected to have all pediatric studies completed by 10 years, only 52.8% had any labeling changes reflecting data from the PREA-mandated studies. A limitation of this study is that publicly available FDA data provide limited detail on study design, execution, and reasons for delays, preventing assessment of study rigor and the factors contributing to delayed completion.

**Conclusions:**

PREA was implemented to advance pediatric drug research and fill a critical gap in pediatric labeling of new drugs. However, our findings reveal frequent delays in study completion and labeling updates, with just over half of labeling additions completed 10 years after drug approval. Strengthening reporting requirements and expanding the FDA’s enforcement authority are essential to ensuring that children receive timely access to safe and effective therapies supported by high-quality evidence.

## Author summary

**Why was this study done?:**

- Many drugs used in children lack pediatric-specific evidence and are prescribed off-label, often based on adult data.
- The Pediatric Research Equity Act (PREA) authorizes the U.S. Food and Drug Administration (FDA) to require pediatric studies for new drugs likely to be used in children.
- It remains unclear how often these required studies are completed and how quickly their findings are reflected in drug labeling.

**What did the researchers do and find?:**

- We analyzed all novel drugs approved by the FDA between 2011 and 2023 to track completion of PREA-required pediatric studies and corresponding updates to drug labels.
- One-third of drugs were subject to pediatric study requirements, but only half of studies were completed, and less than a quarter were finished on time.
- 10 years after approval, just over half of affected drugs had pediatric labeling updates, typically added about six years after approval.

**What do these findings mean?:**

- Despite progress in increasing pediatric drug research under PREA, substantial delays persist in generating and integrating pediatric evidence into drug labels.
- Strengthening transparency, monitoring, and enforcement could help ensure that children benefit from timely, evidence-based drug information.
- Limitations include reliance on public FDA data, which do not explain why studies are delayed, how strong the studies were, or whether they matched what the FDA originally required.

## Abstract 3

In a retrospective analysis of US FDA records of novel, approved drugs with pediatric study requirements, Rylee McGonigle and colleagues report that less than 60% of requirements were met a decade post-approval, and labeling changes were substantially delayed.shorter version: In a retrospective analysis, Rylee McGonigle and colleagues find that post-approval completion of pediatric studies and associated labeling changes are substantially delayed, despite US FDA requirements.

## Introduction

Few novel drugs are developed specifically for pediatric use, leaving many treatments for children dependent on medications studied and approved for adults. Off-label drug use in pediatric populations is common across clinical settings and disease areas, and often guided by clinician experience, consensus statements, or extrapolation from adult data [1–4]. However, this approach relies on a different evidentiary standard than is required for on-label prescribing, where “substantial evidence” of effectiveness and safety, based on “adequate and well-controlled investigations,” is deemed necessary for market approval [5]. The absence of pediatric-specific data can lead to unfavorable benefit-risk profiles, and off-label use has been associated with an increased risk of adverse events as well as ineffective or improperly dosed treatments in children [6–9].

To expand pediatric prescribing information in drug labeling, U.S. Congress enacted the Pediatric Research Equity Act (PREA) in 2003. This law grants the U.S. Food and Drug Administration (FDA) the authority to require sponsors to conduct pediatric studies for certain new drugs when they are deemed relevant to pediatric populations [10]. PREA aims to reduce reliance on non-evidence-based treatments in children by increasing the availability of pediatric data at or near the time of drug approval. Although pediatric development plans are now initiated earlier in the drug development process, many pediatric studies are not completed before market approval and are instead issued as postmarketing requirements [11–13]. However, the agency has limited enforcement tools after drugs are approved and as of 2023, the FDA reported that it had granted over 700 extensions for pediatric study deadlines and 250 studies were categorized as delayed [14,15].

Given that PREA remains the primary regulatory framework for ensuring the safety and efficacy of pediatric drug use in the U.S., assessing its effectiveness is vital for improving pediatric clinical care. Prior research has evaluated PREA-mandated studies within specific cohorts of drug approvals spanning 4- to 8-year periods, focusing on aspects such as study completion rates, results reporting, publication, and transparency [12,13,16,17]. However, given the long durations of pediatric studies and frequent delays [12,13], broader and longer-term analyses are needed to fully assess PREA’s impact on pediatric drug development. Moreover, further evaluation is required to determine whether and when pediatric prescribing information is added to drug labels, which is a central goal of the legislation. In this study, we examined the completion of PREA-mandated pediatric studies and the addition of corresponding pediatric labeling for novel drugs approved by the FDA from 2011 to 2023.

## Methods

### Study sample

For all novel drugs (including new molecular entities and therapeutic biologics) approved by the FDA’s Center for Drug Evaluation and Research (CDER) from 2011 to 2023, we identified the pediatric postmarket studies required by the FDA at the time of original drug approval [18]. Approval letters include a specific section detailing pediatric studies required under PREA, and these were reviewed to identify and collect information on required pediatric studies, including study type, pediatric age groups to be studied, and due dates for submission of study reports to the FDA. While PREA study requirements can be issued also for other types of approvals (e.g., new indications, dosage forms, or routes of administration), we limited our analysis to novel drug approvals to focus on pediatric studies associated with products entering the market during a defined recent time period. Preclinical animal studies were excluded as were studies associated with drugs that were discontinued during the study period, since pediatric studies would no longer be expected for these drugs. All analyses were prespecified, although a written analysis plan is not available. This study is reported as per the Strengthening the Reporting of Observational Studies in Epidemiology (STROBE) guideline (S1 Checklist).

### Data collection on required postmarketing pediatric studies

Using the FDA’s Postmarketing Requirements and Commitments Database, information was collected on the status of studies through December 31, 2024 [19]. Status categories include pending, ongoing, delayed, submitted, fulfilled, and released. Submitted studies are those for which a study report has been provided to the FDA, while fulfilled studies are those for which the study report has been reviewed and approved by the FDA as meeting the terms of the requirement. Studies can be released if the FDA determines that the postmarket study would no longer be feasible or provide useful information. We considered studies to be ongoing if their status was listed as pending, ongoing, or delayed, and completed once they were submitted. For studies that had been submitted, we noted the date of study report submission and considered this as the study completion date. Planned study duration was defined as the time from drug approval to the original study report due date and observed study duration as time from approval to study report submission.

Since information on studies that are fulfilled or released are removed from the database after one year, we consulted archived versions of the database available on the FDA website to obtain information no longer available in the database. For studies with missing information on due dates (*n* = 24) across these sources, we obtained this information from the FDA through a Freedom of Information Act request.

Sponsors can request extensions to study due dates, which if approved by the FDA, result in a new study report due date. Studies can receive multiple extensions and revisions to the study report due date. Information on study extensions granted by the FDA was obtained from a publicly available FDA database that tracks pediatric study extensions for drugs approved by CDER [20]. Studies can also be replaced if the sponsor requests modifications, such as changes to participant age groups or study endpoints. Information on these study replacements, including revised study due dates, was obtained from the FDA Postmarketing Requirements and Commitments Database. In these cases, we noted the study replacement and considered the new study a continuation of the original study requirement. In a sensitivity analysis, study completion was also assessed excluding studies that were replaced to account for potential bias toward higher completion rates.

To supplement the pediatric study descriptions provided in FDA approval letters, we used study records in ClinicalTrials.gov to collect additional information on study primary endpoints. Using the study endpoints, studies were classified into mutually exclusive categories of primary efficacy, safety, or pharmacokinetic/pharmacodynamic (PK/PD) studies. Pediatric age groups were classified as neonates (0–<1 month), infants (1 month–< 2 years), early childhood (2–< 6 years), late childhood (6–< 12 years), and adolescent (12–< 18 years), based on conventional clinical definitions [21]. Therapeutic categories were assigned according to the Anatomic Therapeutic Classification System [22].

### Data collection on addition of pediatric prescribing information to drug labels

For all drugs with an associated pediatric study requirement, we determined whether pediatric labeling changes resulting from the PREA-required studies had been made as of December 31, 2024. Under PREA, sponsors are required to add information generated from the required studies to the drug label, irrespective of the study findings on drug benefit [20]. The FDA maintains a Pediatric Labeling Changes Spreadsheet, which contains information on all pediatric labeling changes made as a result of studies conducted under PREA [23]. We used this data source to identify relevant labeling changes and reviewed the drug labels to determine the type and time to first PREA-specific labeling additions, as well as the pediatric age groups receiving labeling information. A pediatric study was considered to have resulted in labeling changes if any result from the study was added. Since only two drugs in the sample had pediatric studies associated with more than one indication, all labeling additions were considered at the drug level.

### Statistical analysis

We performed descriptive statistics to describe study characteristics, completion of studies, and addition of pediatric prescribing information to drug labels. In assessing study completion and labeling additions, we excluded studies that had not yet reached their expected completion date and limited to the subset of studies with due dates by December 31, 2024. We performed a multivariable linear regression analysis to assess planned study duration, modeling study duration in years as the dependent variable, and including predictors for study type, therapeutic area, and binary indicators for whether a given study included participants from each pediatric age group. Since the pediatric age group indicators are not mutually exclusive and a single study can include participants from multiple age groups, the coefficients for these variables represent the additional association of including each age group while adjusting for the others. We controlled for drug type (small molecule drug versus biologic) and study start year. To account for potential non-independence among studies of the same drug, standard errors were adjusted for clustering by drug. We also assessed the linearity assumption for study start year by comparing a linear specification to a spline-based model; the spline model did not improve fit, supporting the adequacy of treating approval year as a linear term in all the models.

Completion and on-time completion of pediatric studies were evaluated using multivariate logistic regression models, using the same set of predictor and control variables and adjusting for potential non-independence of studies of the same drug. Results are reported as odds ratios with 95% confidence intervals for each level relative to the reference group. In some cases, categories with very sparse data or complete separation (e.g., no events or all events within a group) led to non-convergence of the logistic regression model, and odds ratios could not be reliably estimated.

Among drugs with pediatric labeling additions, differences in mean time to labeling addition across labeling types were assessed using one-way ANOVA. This analysis was restricted to drugs with observed labeling additions, for which all event times were fully observed and uncensored. To examine predictors of time to labeling addition across all drugs (including those without an addition by the study cutoff), we used Cox proportional hazards models. Logistic regression was used to evaluate factors associated with the presence of any pediatric labeling addition. All models were adjusted for drug type and the year of approval. For the Cox proportional Hazards model, the proportional hazards assumption was verified using Schoenfeld residuals, which indicated non-proportionality for approval year. Accordingly, we modeled approval year as a time-varying covariate by including an interaction with log(time).

We used Kaplan–Meier analyses to estimate the cumulative incidences of pediatric study completion and of labeling additions over time. Since the FDA does not set expected dates for labeling additions, the latest expected study completion date among all studies associated with a drug was used as the comparison for the date of labeling addition. Differences between expected and observed study completion dates were evaluated using the Wilcoxon signed-rank test, indicating whether the median observed completion date differed from the expected date. This test was also used to assess differences between the latest expected study completion date associated with a drug and the date of labeling change. Analyses were performed using R Studio Version 2024.12.1 + 563.

### Patient and public involvement

The development of the research question was informed by concerns regarding evidence-based use of therapeutics in pediatric patients. Patients were not involved in the design or conduct of the study, nor were they advisers in this study.

## Results

### Study cohort

There were 552 drugs approved by CDER over the study period, of which 179 (32.4%) were associated with pediatric study requirements under PREA at the time of approval. Thirteen of these drugs were eventually discontinued, yielding a study cohort of 166 drugs associated with a total of 338 pediatric study requirements (mean of 2 study requirements per drug).

### Study characteristics

About half the 338 PREA-required studies were primary efficacy studies (*N* = 175, 51.8%), with another 32.5% (*n* = 110) comprised of primary safety studies, and 15.7% (n = 53) primary PK/PD studies (Table 1). Inclusion in studies increased with increasing pediatric age, with 18.3% (*n* = 62) of studies enrolling neonates and 79.3% (*n* = 268) adolescents. Studies were concentrated across a few therapeutic areas, with almost half studying either nervous system agents (*n* = 92, 27.2%) or anti-infectives (*n* = 70, 20.7%), followed by 15.1% (*n* = 51) focused on alimentary tract drugs.

**Table 1:** Characteristics and planned study durations of required pediatric studies, 2011–2023.

	Pediatric studies, *N* (%)	Planned study duration, mean years (95% CI)	Estimated difference in planned duration, years (95% CI)^1^	Adjusted *p*-value^1^
**All studies**	338	5.6 (5.3, 5.9)		
**Study type**				
Primary efficacy study	175 (51.8)	6.5 (6.0, 6.9)	Referent	
Primary safety study	110 (32.5)	5.3 (4.8, 5.8)	−0.84 (−1.56, −0.13)	0.02
Primary PK/PD study	53 (15.7)	3.2 (2.7, 3.8)	−3.23 (−3.97, −2.49)	<0.01
**Pediatric age group** ^2^				
Neonate (0– <1 month)	62 (18.3)	4.9 (4.3, 5.4)	−0.60 (−1.44, 0.24)	0.16
Infant (1 month– <2 year)	118 (34.9)	5.0 (4.5, 5.5)	0.12 (−0.81, 1.04)	0.81
Early childhood (2–<6 years)	170 (50.3)	5.2 (4.8, 5.6)	−0.63 (−1.43, 0.16)	0.12
Late childhood (6–<12 years)	259 (76.6)	5.6 (5.2, 5.9)	0.63 (−0.28, 1.55)	0.18
Adolescent (12–<18 years)	268 (79.3)	5.5 (5.2, 5.9)	−0.91 (−1.80, −0.01)	0.05
Unspecified	4 (1.2)	6.5 (3.4, 9.5)	2.12 (−0.72, 4.96)	0.14
**Therapeutic area**				
Nervous system	92 (27.2)	6.2 (5.6, 6.8)	Referent	
Anti-infectives for systemic use	70 (20.7)	4.8 (4.3, 5.3)	−1.23 (−2.08, −0.37)	<0.01
Alimentary tract and metabolism	51 (15.1)	6.1 (5.2, 7.0)	−0.81 (−1.85, 0.24)	0.13
Immunomodulating agents	40 (11.8)	6.4 (5.3, 7.6)	0.13 (−1.01, 1.26)	0.83
Antineoplastic agents	17 (5.0)	5.4 (4.3, 6.5)	−0.29 (−1.58, 1.00)	0.66
Hematologic agents	14 (4.1)	3.8 (2.3, 5.3)	−1.42 (−2.68, −0.16)	0.03
Other^3^	54 (16.0)	5.0 (4.3, 5.8)	−1.10 (−1.99, −0.22)	0.01

^1^Adjusted *p*-values were derived from linear regression models controlling for drug type (small molecule drug vs. biologic) and study start year.

^2^Percentages add to greater than 100 since studies can include participants across multiple age groups.

^3^Other includes all therapeutic groups with less than 4% of total study count: Dermatologicals (13, 3.9%), Cardiovascular system (12, 3.6%), Various (12, 3.6%), Respiratory system (6, 1.8%), Systemic hormonal preparations (6, 1.8%), Genitourinary system and sex hormones (4, 1.2%), Antiparasitic products, insecticides and repellents (1, 0.3%).

The mean planned duration for the pediatric studies was 5.6 years (95% CI, 5.3–5.9), with the longest duration seen for efficacy studies at 6.5 years (95% CI, 6.0–6.9), followed by safety studies at 5.3 years (95% CI, 4.8–5.8), and PK/PD studies at 3.2 years (95% CI, 2.7–3.8). There were no significant differences in mean planned duration across the different age groups, though we did observe differences based on the drug’s therapeutic area. Studies for anti-infectives, hematologic agents, and the miscellaneous “other” category showed shorter planned durations compared with nervous system drug studies (differences of −1.23 years [95% CI, −2.08 to −0.37], –1.42 years [95% CI, −2.68 to −0.16], and −1.10 years [95% CI, −1.99 to −0.22], respectively), while other therapeutic areas did not differ significantly.

### Completion of pediatric studies

Of the 338 pediatric studies, 222 had a due date by December 31, 2024, and were included in the analysis examining study completion. Median follow-up time for these studies was 9.1 years (IQR 6.3–11.1). Among the studies, 111 (50.0%) had obtained at least one extension to the study due date, 29 (13.1%) had been replaced by a new study with a later due date, and 126 (56.8%) had either an extension or a replacement resulting in an extended timeline. The mean change to due dates was 2.9 years (SD 2.0).

A total of 122 (55.0%) studies were completed, with 54 (24.3%) completed by the original due date (Table 2). Compared with primary efficacy studies, primary safety studies had higher odds of completion (adjusted OR, 2.45; 95% CI, 1.07–5.62) and primary PK/PD studies showed even greater odds of completion (adjusted OR, 6.49; 95% CI, 2.37–17.80). There was no evidence of differences in study completion across pediatric age groups. For therapeutic area, compared with anti-infectives, studies of nervous system drugs (adjusted OR, 0.34; 95% CI, 0.13–0.85) and immunomodulating agents (OR, 0.18; 95% CI, 0.04–0.79) had lower odds of completion, while other therapeutic areas showed no significant differences. On-time completion did not differ across study types, though with respect to pediatric age groups, those involving early childhood participants had higher odds of on-time completion (adjusted OR, 3.61; 95% CI, 1.49–8.73) compared with studies in neonates. Therapeutic area was also associated with timely completion, with studies for nervous system drugs showing lower odds of on-time completion (adjusted OR, 0.25; 95% CI, 0.08–0.79) compared with studies of anti-infectives. Results were similar in a sensitivity analysis excluding 49 studies that were replaced (S1 Table).

**Table 2:** Completion of required pediatric studies due by December 31, 2024.

	Completed studies, *N* (%)	Adjusted OR^1^ (95% CI)	Studies completed by due date, *N* (%)	Adjusted OR^1^ (95% CI)
**All studies (*n* = 222)**	122 (55.0)		54 (24.3)	
**Study types**				
Primary efficacy study (n = 103)	48 (46.6)	Referent	20 (19.4)	Referent
Primary safety study (n = 70)	39 (55.7)	2.45 (1.07–5.62)	20 (28.6)	1.45 (0.63–3.34)
Primary PK/PD study (*n* = 49)	35 (71.4)	6.49 (2.37–17.80)	14 (28.6)	1.77 (0.71–4.41)
**Pediatric age group^2^**				
Neonate (0–<1 month) (*n* = 49)	25 (51.0)	0.51 (0.19–1.40)	9 (18.3)	0.59 (0.20–1.76)
Infant (1 month–<2 year) (*n* = 87)	53 (60.9)	1.01 (0.38–2.72)	21 (24.1)	0.47 (0.16–1.39)
Early childhood (2–< 6 years) (*n* = 115)	70 (60.9)	2.05 (0.90–4.68)	34 (29.6)	3.61 (1.49–8.73)
Late childhood (6–<12 years) (*n* = 163)	94 (57.7)	0.69 (0.28–1.70)	39 (23.9)	0.46 (0.18–1.15)
Adolescent (12–<18 years) (*n* = 175)	99 (56.6)	1.23 (0.52–2.88)	44 (25.1)	1.79 (0.62–5.17)
Unspecified (*n* = 2)	0 (0.0)	–	0 (0.0)	–
**Therapeutic area**				
Anti-infectives for systemic use (*n* = 61)	39 (63.9)	Referent	22 (36.1)	Referent
Alimentary tract and metabolism (*n* = 37)	21 (56.8)	0.86 (0.30–2.44)	4 (10.8)	0.25 (0.08–0.79)
Nervous system (*n* = 57)	23 (40.4)	0.34 (0.13–0.85)	12 (21.1)	0.39 (0.15–1.03)
Immunomodulating agents (*n* = 15)	6 (40.0)	0.18 (0.04–0.79)	0 (0.0)	–
Antineoplastic agents (*n* = 3)	3 (100.0)	–	1 (33.3)	0.53 (0.01–22.37)
Hematologic agents (*n* = 11)	7 (63.6)	0.88 (0.22–3.49)	3 (27.3)	0.68 (0.13–3.45)
Other (*n* = 38)	23 (60.5)	0.89 (0.29–2.69)	12 (31.6)	0.71 (0.25–2.05)

^1^Adjusted odds ratios were derived from logistic regression models controlling for drug type (small molecule drug vs. biologic) and study start year.

^2^The sample of studies for each age group represents the studies enrolling pediatric participants in that age group. Studies can be included in multiple age groups depending on the age range eligible for enrollment.

**Fig 1:**
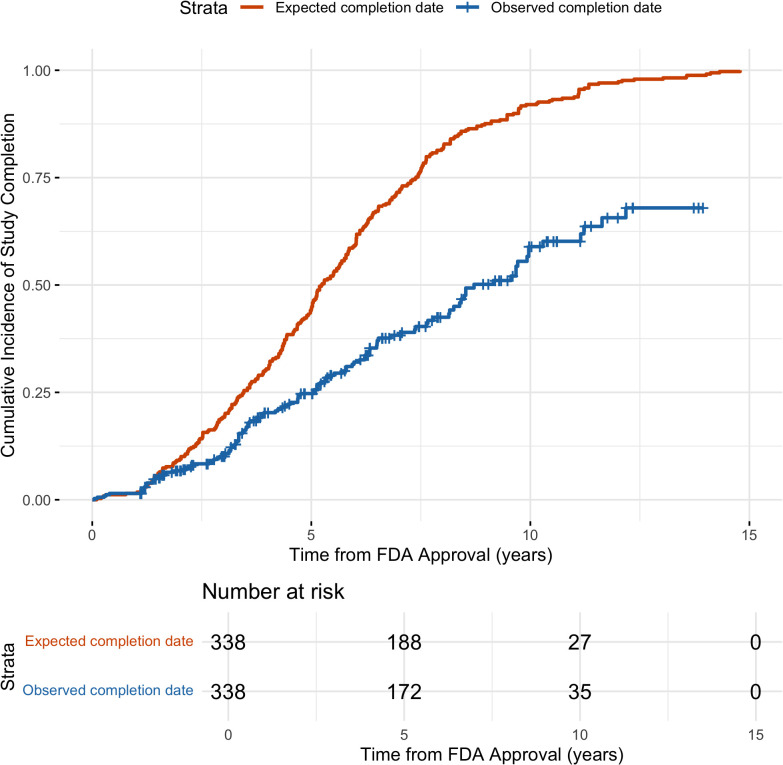
Time to completion of pediatric studies required for novel drugs. Kaplan–Meier curves were used to assess the cumulative incidence of study completion for pediatric studies required under PREA, with curves illustrating cumulative incidence of completion based on expected and on observed study completion dates.

Fig 1 shows Kaplan–Meier curves for the cumulative incidence of study completion based on expected and observed completion dates. By 5 years after drug approval, 44.4% of pediatric studies were expected to be completed based on the original due date, compared with 24.8% observed to be completed. This difference increased over time and by 10 years, 92.0% of studies were expected to be completed, but only 59.5% were completed. Overall, studies were completed significantly later than expected (Wilcoxon signed-rank test, *p* = 0.01), indicating systematic delays relative to planned timelines.

### Addition of pediatric prescribing information

The 222 pediatric studies due by December 31, 2024, corresponded to 117 drugs (Table 3). Just over half of these drugs (*n* = 64, 54.7%), had pediatric information added by December 31, 2024, with a mean of 5.6 years (SD 2.7) to the addition of labeling information. The most common type of labeling change was the addition of approval for pediatric use (*n* = 45/64, 70.3%), followed by expansion of the approved pediatric age range (*n* = 12/64, 18.7%), and addition of information describing lack of safety and effectiveness in pediatric patients (*n* = 7/64, 10.9%). There were significant differences in time to labeling additions based on type of labeling change (ANOVA, *p* < 0.001), with the shortest duration observed for expansion of approved pediatric age ranges at a mean of 3.8 years (SD 1.3), followed by new pediatric approval at 5.7 years (SD 2.6), and addition of information on lack of pediatric safety and efficacy, which took a mean of 8.3 years (SD 3.3).

**Table 3:** Drugs with pediatric labeling additions resulting from required pediatric studies due by December 31, 2024.

	Drugs with labeling additions, *N* (%)	Adjusted OR^1^ (95% CI)	Time to labeling addition, mean years (SD)	Adjusted HR^2^ (95% CI)
**All drugs (*n* = 117)**	64 (54.7)		5.6 (2.7)	
**Pediatric age group^3^**				
Neonate (0–<1 month) (*n* = 29)	15 (51.7)	0.39 (0.07–2.00)	5.3 (1.9)	0.90 (0.39–2.04)
Infant (1 month–<2 year) (*n* = 51)	30,58.8)	0.64 (0.11–3.96)	5.0 (2.4)	0.65 (0.27–1.61)
Early childhood (2–< 6 years) (*n* = 65)	39 (60.0)	3.18 (0.71–15.23)	5.1 (2.5)	1.60 (0.73–3.50)
Late childhood (6–< 12 yrs) (*n* = 100)	55 (55.0)	0.75 (0.10–5.05)	5.9 (2.8)	0.89 (0.34–2.31)
Adolescent (12–< 18 years) (*n* = 107)	58 (54.2)	0.44 (0.04–4.03)	5.8 (2.8)	0.37 (0.12–1.18)
**Therapeutic areas**				
Anti-infectives for systemic use (*n* = 30)	22 (73.3)	Referent	4.3 (1.6)	Referent
Alimentary tract and metabolism (*n* = 18)	9 (50.0)	0.08 (0.01–0.42)	9.2 (3.3)	0.16 (0.07–0.40)
Nervous system (*n* = 23)	8 (34.8)	0.13 (0.02–0.70)	5.3 (2.3)	0.22 (0.09–0.55)
Immunomodulating agents (*n* = 12)	4 (33.3)	0.03 (0.00–0.25)	6.9 (1.6)	0.07 (0.02–0.25)
Antineoplastic agents (*n* = 3)	1 (33.3)	0.08 (0.00–9.01)	5.9 (0)	0.17 (0.02–1.47)
Hematologic agents (*n* = 5)	3 (60.0)	0.30 (0.03–3.43)	6.9 (3.1)	0.44 (0.12–1.54)
Other (*n* = 26)	17 (65.4)	0.51 (0.09–2.61)	5.2 (2.3)	0.45 (0.23–0.91)

^1^Adjusted odds ratios were derived from logistic regression models adjusting for drug type (small molecule drug vs. biologic) and study start year.

^2^Adjusted hazard ratios were derived from Cox proportional hazards models adjusting for drug type (small molecule drug vs. biologic) and study start year.

^3^The sample for each age group represents the drugs with studies enrolling pediatric participants in that age group. Drugs can be included in multiple age groups depending on the age range eligible for enrollment.

The likelihood of a pediatric labeling addition did not differ by age group. In contrast, labeling additions varied substantially by therapeutic area. Compared with anti-infectives, drugs in the alimentary tract and metabolism (adjusted OR, 0.08; 95% CI, 0.01–0.42), nervous system (OR, 0.13; 95% CI, 0.02–0.70), and immunomodulating agent (OR, 0.03; 95% CI, 0.00–0.25) classes had lower odds of receiving a pediatric labeling addition. Similarly, in time-to-event analyses, the rate of pediatric labeling additions did not differ across age groups, though differences were seen based on therapeutic area. Drugs in the alimentary tract and metabolism (adjusted HR, 0.16; 95% CI, 0.07–0.40), nervous system (HR, 0.22; 95% CI, 0.09–0.55), and immunomodulating agent (HR, 0.07; 95% CI, 0.02–0.25) classes had slower rates of pediatric labeling additions compared to anti-infective drugs.

**Fig 2:**
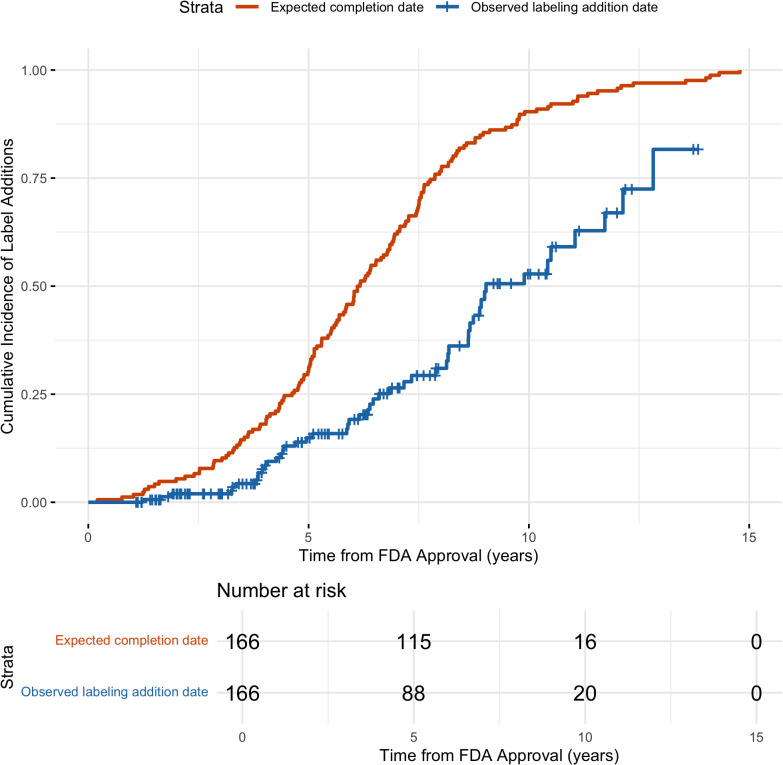
Time to addition of pediatric prescribing information for novel drugs. A Kaplan–Meier curve was used to assess cumulative incidence of drugs with pediatric labeling additions resulting from pediatric studies required under PREA. For comparison, a Kaplan–Maier curve is also shown for the cumulative incidence of study completion based on the latest expected completion date among all studies associated with a drug.

Kaplan–Meier curves are shown in Fig 2 for the cumulative incidence of expected pediatric study completion and observed labeling additions over time. At 5 years after drug approval, 30.7% of drugs were estimated to have had all pediatric studies completed, but only 14.9% of drugs had labeling changes. By 10 years, 90.4% of drugs were expected to have had all pediatric studies completed, but only 52.8% of drugs had pediatric information added to labels. Comparison of the latest planned study completion date with the date of labeling changes indicated that labeling updates occurred significantly later than expected (Wilcoxon signed-rank test, *p* = 0.04).

## Discussion

In this analysis of novel drugs approved by the FDA from 2011 to 2023, approximately one-third were subject to postmarketing study requirements under PREA. Despite the intent of these requirements to increase the timely availability of pediatric prescribing information, we found that fewer than 25% of studies were completed by their original due dates, and even 10 years after approval, less than 60% had been completed. There were consequently delays in the addition of pediatric labeling information: just over half of drug labels included pediatric data a decade after drug approval. For these drugs, it took six years for pediatric approvals to be added, and eight years for labels to reflect findings that demonstrated lack of efficacy or safety in pediatric patients.

These prolonged timelines highlight the need for targeted efforts to identify and address barriers that can limit the generation of high-quality evidence to inform pediatric use. Conducting drug trials in children presents unique challenges, including small and heterogeneous patient populations that often make it difficult to enroll sufficient numbers of participants across relevant age ranges [24,25]. Ethical and feasibility concerns further complicate study design, particularly with regard to minimizing risk, obtaining informed consent and assent, and balancing clinical equipoise [26]. In addition, enrollment in postmarketing studies may be particularly challenging, as families and clinicians may be reluctant to participate in randomized trials if drug access outside of the research setting is already readily attainable [27,28]. These factors warrant careful consideration in the planning and design of PREA studies to ensure feasibility and timely completion. In the current analysis, the average planned duration of PREA studies was around 6 years, which is considerably longer than the 2–3 years reported for adult postmarking studies, though these adult studies are nonetheless more likely to be completed on-time [29–31]. Reducing study delays will require consideration beyond the prescribed timeframes to focus on disease-specific study design and preparation, clinical research infrastructure needs, sponsor incentives, and sponsor readiness in initiating and conducting studies [32].

This study builds upon prior research by using an extended time frame and linking study requirements to labeling changes, enabling a more comprehensive evaluation of how PREA-mandated studies translate into clinical practice. Leveraging the large study sample, we focused on studies with due dates though December 31, 2024, to estimate an overall completion rate of 55% based on a median follow-up of 9.1 years. This is in contrast to results of a recent study examining studies issued from 2015 to 2021, which found a 28% completion rate after a median follow-up of 5.6 years [17]. The discrepancy reflects differences in follow-up periods and underscores the need for standardized metrics to assess pediatric study activity. Our Kaplan–Meier analysis revealed a 5-year completion rate of 25%, similar to the results from this recent study as well as to the rate of 27% reported in an earlier study using the same methods as the current analysis to examine drugs approved from 2007 to 2014 [12]. This approach also allowed us to compare observed timelines with projected study durations, providing benchmarks to assess compliance and identify gaps.

Despite the central goal of PREA to improve pediatric labeling, this outcome remains understudied. A prior analysis of drugs approved from 2003 to 2012 found a mean time of 6.5 years from approval to pediatric study submission, but did not examine time to labeling changes or their content [13]. Another study, focused on approvals from 2007 to 2014, reported that 30% of drugs initially approved without pediatric information had their labels updated based on required studies after a median of 7 years [12]. This is consistent with our findings, with Kaplan–Meier estimates indicating that 15% of drugs had labeling updates at 5 years, increasing to 53% at 10 years.

Our analysis also provides insight into variation by pediatric age group and therapeutic area. Over half of studies included patients under 6 years old. While studies in younger children are often considered more difficult, we did not find significant differences in the likelihood of study completion based on patient age groups enrolled in studies. We also did not observe differences in labeling changes or time to labeling changes across age groups, though given small sample sizes for certain age groups, ongoing monitoring will be important to better understand age-specific challenges.

Notable differences were observed across therapeutic areas. Drugs such as alimentary tract and immunomodulating agents had particularly long planned study durations, with average study durations exceeding 6 years. These drugs also had the lowest rates of on-time study completion (10.8% and 0%, respectively) and the longest delays to labeling updates (9.2 years and 6.9 years, respectively). While insufficient information was available to further assess underlying factors, identifying variation by drug type can inform future oversight strategies and guide efforts to ensure timely pediatric research.

Our findings affirm the value of the PREA legislation: a significant proportion of drugs developed for adults are deemed relevant to pediatric populations and are approved with pediatric study requirements. Nonetheless, there are several shortcomings in how the framework is implemented that may undermine its effectiveness in reducing off-label and non-evidence-based drug use in children.

One critical issue is the lack of timely, detailed, reporting on pediatric study progress and outcomes. Although the FDA is required to report pediatric labeling changes resulting from PREA [20], existing reports are often outdated and lack contextual detail. For example, the agency’s “Annual Pediatric Labeling Changes” report provides annual totals by type of change through 2021 [33], but does not specify how many changes were expected, the time to labeling change, which pediatric age groups were affected, or how many drugs still lack adequate pediatric information. Without this context, such reports are of limited utility in evaluating progress or identifying areas for improvement. More comprehensive reporting—linking study requirements, progress, and labeling changes to specific drugs—would improve transparency, support oversight, and facilitate regulatory enforcement.

Transparency is also lacking around the design and execution of pediatric studies. While study requirements are described in approval letters, the information is often limited and inconsistent, with few details on endpoints, sample sizes, or the rationale behind study designs [12,16]. Additionally, while sponsors frequently request extensions to study deadlines—and approximately 70% of these are granted by the FDA [14]—there is little public information on the reasons for these delays or the FDA’s evaluation of such requests. The FDA’s deferral tracking sheet contains only vague justifications, such as “delays involving study participants, sites, and/or management” or “additional time required to prepare the study report and/or submission” [20]. In some cases, study requirements are withdrawn altogether, without explanation. For example, the FDA approved rolapitant in 2015 for use in adults with chemotherapy-induced nausea and vomiting and initially required two pediatric studies. These were modified in 2019 and then released in 2020 without replacement. Understanding the rationale for such changes—especially when related to scientific concerns—would support clinician decision-making and strengthen future regulatory strategies.

Another important issue is the prioritization of pediatric study requirements to maximize their feasibility and public health value. Given the challenges of enrolling children in clinical research and the long timelines required for study completion, ensuring that requirements focus on studies most likely to generate clinically meaningful evidence is essential [11]. The current system does not account for competing pediatric enrollment needs when there are multiple drug development programs within a given therapeutic area, potentially leading to failures to meet enrollment targets across study requirements [34]. A data-driven approach to prioritization should account for therapeutic need in pediatric populations, disease prevalence, feasibility of study conduct, and scientific considerations, such as the potential to extrapolate findings from adults or inclusion of certain pediatric groups in later-phase adult studies [35]. Aligning requirements with both clinical relevance and operational feasibility could help reduce delays and accelerate the availability of evidence to guide pediatric prescribing.

Additional enforcement mechanisms are also needed to ensure compliance. While the FDA can issue noncompliance letters for overdue pediatric study reports, it is unclear how consistently these are issued or how much impact they have on sponsor behavior. A notable limitation is that the letters can be issued only after the final report deadline has been missed, even though the FDA tracks study progress throughout trial conduct and is often aware of delays and missed milestone deadlines long before the study completion due date. Another consideration around enforcement is that unlike for adult postmarket requirements, PREA specifically prohibits the FDA from imposing monetary penalties [36]. In 2023, the U.S. House of Representatives introduced legislation to remove this exemption and strengthen the FDA’s enforcement authority [37]. A similar bill was introduced in the Senate in 2024 [38]. Although neither bill was enacted, the bipartisan support for the amendment is encouraging and signals a growing consensus on the need to improve regulatory tools and ensure timely completion of required pediatric studies.

This study has several limitations. Although PREA applies to a range of application types—including new active ingredients, indications, dosage forms, dosing regimens, and routes of administration—we focused only on new active ingredients and associated indications. As a result, we may not have captured all pediatric studies required by the FDA for a given drug. Additionally, pediatric postmarketing studies mandated under other authorities were not included, though PREA remains the primary mechanism for requiring pediatric research. Despite the long study period and large sample size, there were few observations for certain subgroups, limiting statistical power to detect differences. Furthermore, due to limited detail on study design, we could not assess the rigor of the proposed studies nor how closely the final studies aligned with original requirements. We also did not have additional information on study sponsors or contract research organizations and could not account for potential non-independence among studies conducted by the same entities. Finally, the causes of study delays remain unclear, as public data lack sufficient information to identify contributing factors. These limitations underscore the importance of increased transparency and improved monitoring of the FDA’s oversight of research for pediatric drug approvals.

More than two decades after its passage, PREA remains a cornerstone of pediatric drug regulation in the United States. However, our findings reveal substantial delays in study completion and labeling updates, as well as significant gaps in transparency and enforcement. To address these issues, policymakers and regulators should consider targeted reforms aimed at improving reporting, increasing transparency around study design and delays, and strengthening the FDA’s enforcement authority. These efforts are essential to advancing pediatric drug development and ensuring that children receive safe and effective therapies based on robust evidence.

## Supporting information

S1 Checklist STROBE checklist. https://www.strobe-statement.org/checklists/ licensed under CC BY 4.0 (https://creativecommons.org/licenses/by/4.0/).
(DOCX)

S1 Table Sensitivity analysis. Sensitivity analysis examining completion of required pediatric studies due by December 31, 2024, excluding 49 studies that were replaced.
(DOCX)

## Abbreviations

CDER: Center for Drug Evaluation and Research
FDA: Food and Drug Administration
PK/PD: pharmacokinetic/pharmacodynamic
PREA: Pediatric Research Equity Act
