# Supplementary material for: Pediatric studies and labeling additions required by the U.S. FDA for novel drugs approved from 2011 to 2023: A retrospective cohort study
Source: PLoS Med. 2025 Dec 17;22(12):e1004651. doi: 10.1371/journal.pmed.1004651 (PMC12742784; doi:10.1371/journal.pmed.1004651)
Supplement: S1 Table — Sensitivity analysis examining completion of required pediatric studies due by December 31, 2024, excluding 49 studies that were replaced. (DOCX) [file pmed.1004651.s002.docx]

**S1 Table. Sensitivity analysis examining completion of required pediatric studies due by December 31, 2024, excluding 49 studies that were replaced**

|  | **Completed studies, N (%)** | **Adjusted OR (95% CI)** | **Studies completed by due date, N (%)** | **Adjusted OR (95% CI)** |
| --- | --- | --- | --- | --- |
| **All studies (n=173)** | 98 (56.6) |  | 45 (26.0) |  |
| **Study types** |  |  |  |  |
| Primary efficacy study (n=73) | 34 (46.6) | Referent | 16 (21.9) | Referent |
| Primary safety study (n=58) | 33 (56.9) | 2.06 (0.77–5.52) | 16 (27.6) | 1.13 (0.44–2.89) |
| Primary PK/PD study (n=42) | 31 (73.8) | 9.44 (3.05–29.20) | 13 (31.0) | 2.12 (0.72–6.23) |
| **Pediatric age group^1^** |  |  |  |  |
| Neonate (0 - <1 month) (n=35) | 19 (54.3) | 0.35 (0.09–1.38) | 6 (17.1) | 0.33 (0.08–1.34) |
| Infant (1 month - <2 year) (n=67) | 44 (65.7) | 1.24 (0.37–4.18) | 17 (25.3) | 0.59 (0.17–2.05) |
| Early childhood (2 - <6 years) (n=89) | 58 (65.2) | 2.79 (1.04–7.50) | 29 (32.6) | 3.64 (1.31–10.09) |
| Late childhood (6 - <12 years) (n=126) | 76 (60.3) | 0.76 (0.25–2.34) | 33 (26.2) | 0.48 (0.16–1.41) |
| Adolescent (12 - <18 years) (n=139) | 79 (56.8) | 0.94 (0.31–2.90) | 36 (25.9) | 1.47 (0.43–5.05) |
| Unspecified (n=2) | 0 (0) | - | 0 (0) | - |
| **Therapeutic area** |  |  |  |  |
| Antiinfectives for systemic use (n=46) | 31 (67.4) | Referent | 19 (41.3) | Referent |
| Alimentary tract and metabolism (n=27) | 16 (59.3) | 0.61 (0.15–2.48) | 3 (11.1) | 0.13 (0.03–0.52) |
| Nervous system (n=45) | 18 (40.0) | 0.23 (0.07–0.78) | 9 (20.0) | 0.22 (0.07–0.72) |
| Immunomodulating agents (n=10) | 3 (30.0) | 0.11 (0.01–0.85) | 0 (0) | - |
| Antineoplastic agents (n=2) | 2 (100.0) | - | 0 (0) | - |
| Hematologic agents  (n=11) | 7 (63.6) | 0.56 (0.12–2.60) | 3 (27.3) | 0.44 (0.08–2.33) |
| Other (n=32) | 21 (48.8) | 0.96 (0.26–3.48) | 11 (34.4) | 0.48 (0.14–1.67) |

^1^ The sample of studies for each age group represents the studies enrolling pediatric participants in that age group. Studies can be included in multiple age groups depending on the age range eligible for enrollment.
